# BDNF impact on synaptic dynamics: extra or intracellular long-term release differently regulates cultured hippocampal synapses

**DOI:** 10.1186/s13041-020-00582-9

**Published:** 2020-03-17

**Authors:** Rossana Rauti, Giada Cellot, Paola D’Andrea, Andrea Colliva, Denis Scaini, Enrico Tongiorgi, Laura Ballerini

**Affiliations:** 1 Life Science Department, University of Trieste, Via Giorgieri, 5 Build Q, 34127 Trieste, Italy; 2 International School for Advanced Studies (SISSA), Via Bonomea 265, 34136 Trieste, Italy; 3 Present address: Department of Biomedical Engineering, Tel-Aviv University, Tel-Aviv, Israel; 4 Present address: Cardiovascular Biology, International Centre for Genetic Engineering and Biotechnology (ICGEB), Trieste, Italy; 5 ELETTRA Synchrotron Light Source, 34149 Trieste, Italy

**Keywords:** Neurotrophin, Hippocampal network, Patch-clamp, Synaptic activity, Miniature synaptic current

## Abstract

Brain Derived Neurotrophic Factor (BDNF) signalling contributes to the formation, maturation and plasticity of Central Nervous System (CNS) synapses. Acute exposure of cultured brain circuits to BDNF leads to up-regulation of glutamatergic neuro-transmission, by the accurate tuning of pre and post synaptic features, leading to structural and functional synaptic changes. Chronic BDNF treatment has been comparatively less investigated, besides it may represent a therapeutic option to obtain rescue of post-injury alterations of synaptic networks. In this study, we used a paradigm of BDNF long-term (4 days) incubation to assess in hippocampal neurons in culture, the ability of such a treatment to alter synapses. By patch clamp recordings we describe the augmented function of excitatory neurotransmission and we further explore by live imaging the presynaptic changes brought about by long-term BDNF. In our study, exogenous long-term BDNF exposure of post-natal neurons did not affect inhibitory neurotransmission. We further compare, by genetic manipulations of cultured neurons and BDNF release, intracellular overexpression of this neurotrophin at the same developmental age. We describe for the first-time differences in synaptic modulation by BDNF with respect to exogenous or intracellular release paradigms. Such a finding holds the potential of influencing the design of future therapeutic strategies.

## Introduction

Brain-derived neurotrophic factor (BDNF) is a member of the neurotrophin family crucial to brain and spinal cord development [1, 2]. Exposure to BDNF during development stimulates axonal branching [3, 4], dendritic growth [4, 5], and refinement of synapses in an activity-dependent manner [6]. In addition, BDNF promotes the formation of both excitatory and inhibitory synapses and increases their maturation [7].

Among the members of the neurotrophin family, BDNF stands out for its ability to regulate synaptic plasticity and various cognitive functions of the brain [8]. Indeed, BDNF, when acutely delivered, has been shown to affect synaptic transmission and plasticity [9–11]. In particular, a rapid enhancement of excitatory neurotransmission has been demonstrated in dissociated hippocampal cultures [12–16] and in cultured neuromuscular synapses [17–19]. BDNF modulates also inhibitory transmission, albeit through different mechanisms [20, 21]. BDNF potentiates excitatory synapses via pre- and post-synaptic mechanisms [22, 23]. Presynaptically, BDNF increases glutamate release, thus enhancing the frequency of miniature excitatory postsynaptic currents (mEPSCs) in hippocampal neurons [16, 24]. On the postsynaptic side, BDNF augments NMDA receptor single channel opening probability [15, 25–27]. In addition, BDNF up-regulates the expression of voltage-gated Ca^2+^ and Na^+^ channels at the plasmamembrane [4]. Regarding the inhibitory synaptic transmission, BDNF is needed to regulate maturation of γ-aminobutyric acid (GABAergic) synapses in the hippocampus [28, 29]. Acute BDNF application reduces inhibitory synaptic transmission in the hippocampus, where both evoked and spontaneous GABAergic currents are decreased via TrkB receptor [30, 31]. Finally, acute application of the mature form of BDNF on hippocampal slices facilitates the early phase of long-term potentiation (LTP [32–34];).

Of note, the role of chronic BDNF exposure in regulating long-lasting changes in synaptic function has been comparably less investigated. It was shown that long-term treatment (72 h) of hippocampal slices with BDNF (250 ng/mL) increases synapse number and spine density at the level of apical dendrites of pyramidal neurons in the hippocampus [35], suggesting that BDNF acts selectively on different types of spines, depending on the level of basal, spontaneous synaptic transmission. In addition, it was suggested that long-lasting BDNF treatment might play a role on post injury alteration of synaptic networks and neuronal rescue [10]. Although elevated levels of BDNF have been shown to persist for several days following nerve injury [36–38], the large majority of studies examining its action on dorsal horn neurons have used acute, short-term exposures [39–41]. In this study, we used hippocampal cultures, electrophysiology, live imaging, immunofluorescence and genetic transfections to fill the existing gap of information on long-term actions of BDNF on synaptic dynamics.

## Results

### Long-term BDNF treatment improves excitatory synaptic current frequency and amplitude

**Fig. 1:**
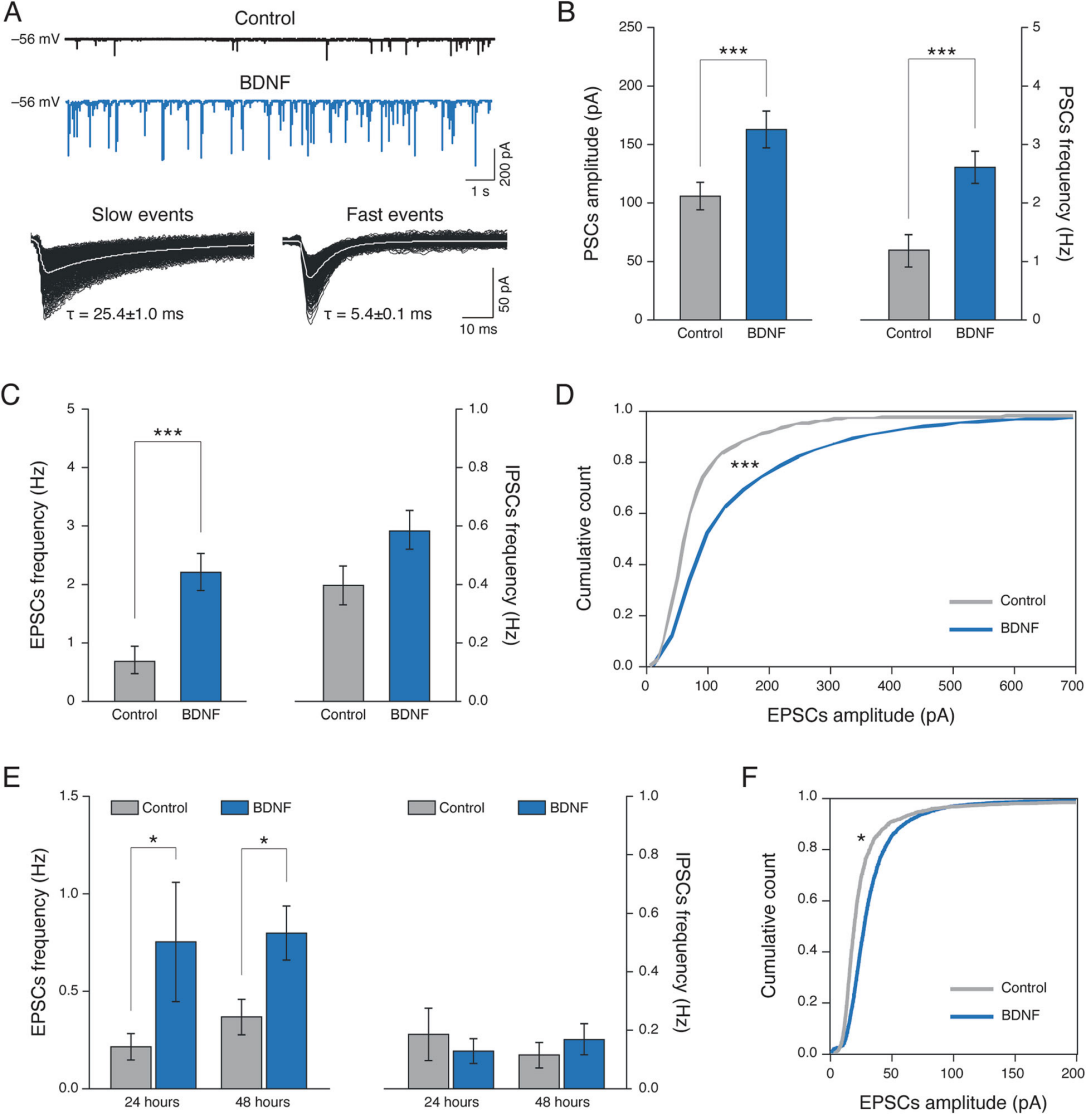
Long-term BDNF exposure augments glutamatergic excitatory synaptic currents. In (**a**) top panel: voltage clamp recordings of spontaneous synaptic activity of Control (black trace) and BDNF treated (blue trace) cultures; bottom panel: average tracings of spontaneous GABA_A_ receptor (slow events; left) and AMPA-glutamate (fast events; right) receptor mediated PSCs, note the different decay time. In (**b**) bar plots reporting PSCs amplitude (left) and frequency (right), significantly (*P* < 0.001, one-way ANOVA) increased by BDNF exposure. In (**c**) bar plots reporting EPSCs (left; significantly increased when compared to Control; *P* < 0.001, one-way ANOVA) and IPSCs (right) frequency following BDNF treatment. Note the different scale of EPSCs and IPSCs in the frequency plot. In (**d**) cumulative distribution of amplitude values of Control (grey) and BDNF (blue) EPSCs (Kolmogorov-Smirnov test; *P* < 0.001). In (**e**) plots reporting EPSCs frequency (left) increased (*P* < 0.05, Mann-Whitney test) after 24 and 48 h long lasting BDNF treatments respect to Controls, while IPSCs frequency (right) is unchanged. In (**f**) cumulative distribution of amplitude values of Control (grey) and BDNF (blue) EPSCs after 24–48 h (*P* < 0.0045 Kolmogorov-Smirnov’s test)

To investigate the ability of prolonged exposure to BDNF to regulate network dynamics and synaptic transmission, dissociated hippocampal cultures were treated for 4 days with 20 nM mature BDNF (named BDNF-treated cultures) and compared to untreated ones (named Controls). Spontaneous postsynaptic currents (PSCs) were recorded using single cell patch-clamp electrophysiology to monitor the activity of the networks grown for 8~10 days in Control and BDNF-treated cultures. The appearance of PSCs provides clear evidence of functional synapse formation [42, 43] and we selected a time in vitro at which synaptic currents are present, but characterized by a relatively low frequency [44, 45]. Figure 1a, (top), shows representative current tracings depicting the basal synaptic activity in cultured neurons. Neuronal passive properties were routinely measured in Control neurons (*n* = 44) and BDNF-treated ones (*n* = 75) which did not differ in terms of cell capacitance (78 ± 5 pF in Control and 96 ± 6 pF in BDNF-treated), input resistance (500 ± 53 MΩ Control and 477 ± 47 MΩ) and resting membrane potential (− 47 ± 1 mV for Control and − 50 ± 1 mV for BDNF-treated). Conversely, in BDNF-treated neurons, baseline PSCs displayed a significant increase (*P* < 0.001; one-way ANOVA) in amplitude (from 111.9 ± 13.3 pA in Controls to 157.2 ± 19.1 pA in BDNF cultures; *n* = 19 and *n* = 26, respectively; summarized in the bar plot of Fig. 1b, left) and in frequency (from 1.2 ± 0.3 Hz in Controls to 2.7 ± 0.3 Hz in BDNF-treated; Fig. 1b, right). In cultured hippocampal networks, neurons typically display either GABA_A_ or glutamate AMPA receptor-mediated PSCs [46, 47], which were both detected as inward currents in our recording conditions (see methods) [42, 45]. Thus, we identified the different populations of PSCs on the basis of their kinetic properties and pharmacology [42, 48]. GABAergic PSCs were identified by their slow decay time constant (τ = 25.4 ± 1.0 ms, *n* = 45; Fig. 1a, bottom left), inverted polarity at − 35 mV holding potential and were abolished by 10 μM bicuculline (*n* = 25). Glutamatergic AMPA receptor-mediated PSCs were identified by their fast decay (τ = 5.4 ± 0.2 ms, *n* = 46; Fig. 1a, bottom right), inverted polarity around 0 mV holding potential and were completely removed by 10 μM CNQX (*n* = 30). In BDNF treated cultures, the frequency of fast excitatory PSCs (EPSCs) was significantly increased when compared to Controls (from 0.88 ± 0.24 Hz in Control to 2.16 ± 0.31 Hz in BDNF-treated; *n* = 24 and *n* = 38, respectively; *P* < 0.001 one-way ANOVA, Fig. 1c, left) as well as their amplitude (from 74.2 ± 1.4 pA in Control to 146.4 ± 2 pA in BDNF-treated; *P* < 0.001 Mann-Whitney’s test; the cumulative probability distribution of EPSCs amplitudes is shown Fig. 1d; *P* < 0.001 Kolmogorov-Smirnov’s test). Differently, not significant differences were detected in GABA_A_ receptor mediated PSCs (IPSCs) frequency (from 0.40 ± 0.08 Hz in Control to 0.60 ± 0.08 Hz in BDNF-treated; *n* = 21 and *n* = 36, respectively; *P* = 0.19 Student’s t-test; Fig. 1c, right) or peak amplitude (from 152 ± 6 pA Control, to 182 ± 4 pA BDNF-treated; *P* = 0.45 one-way ANOVA) when comparing BDNF-treated with respect to Control.

In subsequent experiments, we tested BDNF exogenous applications at two shorter incubation times, 24 and 48 h, to explore whether, also in these conditions, the prolonged exposure to the neurotrophins was selectively targeting glutamatergic synapses, leaving GABAergic ones unaffected. In BDNF-treated cultures at 24 h and at 48 h the frequency of EPSCs was significantly increased, with respect to Control (at 24 h: from 0.21 ± 0.06 Hz Control to 0.75 ± 0.31 Hz BDNF-treated, *n* = 6 cells each group, *P* = 0.023 Mann-Whitney’s test; at 48 h: from 0.36 ± 0.09 Hz Control to 0.80 ± 0.14 Hz BDNF-treated, *n* = 6 cells each group, *P* = 0.015 Mann-Whitney’s test; Fig. 1e, left). Conversely, the frequency of IPSCs remained unchanged (at 24 h: 0.19 ± 0.09 Hz Control and 0.17 ± 0.05 Hz BDNF-treated, *P* = 0.59 Student’s t-test; at 48 h: 0.12 ± 0.04 Hz Control and 0.17 ± 0.05 pA BDNF-treated, *P* = 0.46 Student’s t-test; Fig. 1e right). In Fig. 1f, the cumulative probability distribution (24 h and 48 h were pooled together; *P* < 0.0045 Kolmogorov-Smirnov’s test) shows the increase in EPCSs peak amplitude following BDNF treatments when compared to Control (from 29 ± 1 pA Control to 36 ± 1 pA BDNF-treated, *P* < 0.0001, Mann-Whitney’s test). Conversely, IPSCs peak amplitudes were unchanged (77 ± 4 pA Control and 64 ± 4 pA BDNF-treated, *P* = 0.065, Mann-Whitney’s test).

### Long-term BDNF treatment affects neuronal survival and synaptic components

**Fig. 2:**
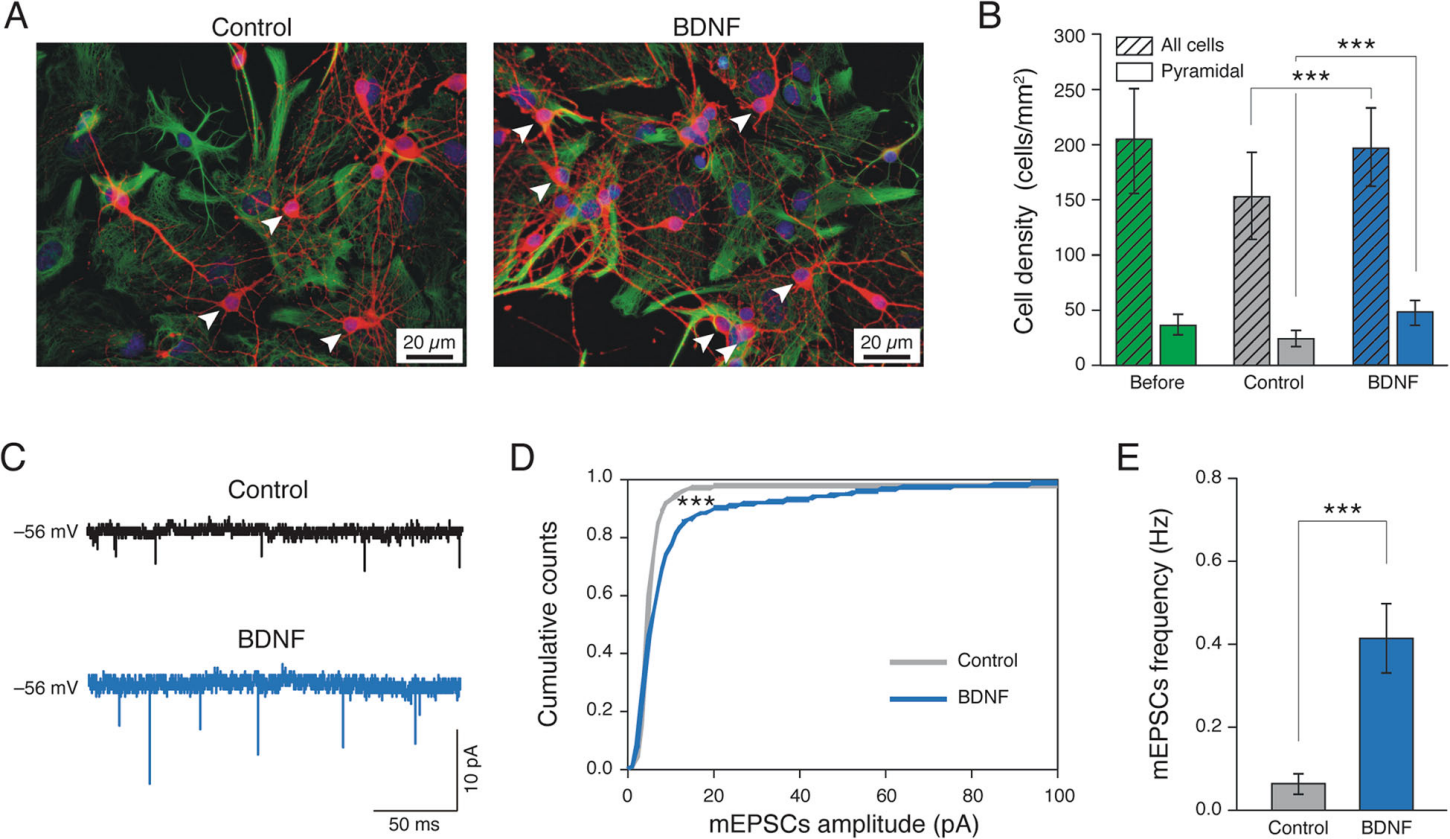
Long-term BDNF exposure impacts neuronal survival and excitatory synapses. In (**a**) Immunofluorescence images showing neuronal and glial cells in Control (left) and BDNF (right) conditions (anti-β-tubulin III, in red; anti-GFAP, in green; in all, nuclei are visualized by DAPI in blue). In (**b**) the plots summarize neuronal densities and pyramidal cells density on Control (in grey) and after BDNF exposure (in blue) compared to cellular densities before any treatment (in green); striped bars refer to all neuronal cells while plain bars refer to pyramidal ones. In (**c**) sample tracings of mEPSCs recorded in Control (black trace) and BDNF (blue trace) treated cultures. In (**d**) cumulative distribution of mEPSCs amplitude values of Control (grey) and BDNF (blue) EPSCs (Kolmogorov-Smirnov test; *P* < 0.001). In (**e**) bar plots reporting mEPSC frequency values. BDNF treatment significantly increased the frequency of mEPSCs (*P* < 0.001, one-way ANOVA)

Neural network efficacy depends on neurons and synapses. Thus, to estimate changes in these structural entities we evaluated the dynamic of network size during development in the two culture groups, since neuronal cell density might influence network activity. We used immunofluorescence to image the specific cytoskeletal components β-tubulin III to identify neurons, and glial fibrillary acidic protein (GFAP) to visualize astrocytes (Fig. 2a). We sampled cultures prior to BDNF incubation by measuring neuronal densities and estimating the number of pyramidal cells identified by morphological criteria (see methods; Fig. 2a, white arrows) [49–51]. β-tubulin III positive cell density before treatment was 208 ± 55 neurons/mm^2^, with 34 ± 7 pyramidal cells/mm^2^ (*n* = 10 visual fields, four different series of cultures; bar plot of Fig. 2b). After 4 days, we detected 154 ± 51 neurons/mm^2^ (25 ± 6 pyramidal cells/mm^2^) in Control and 196 ± 49 neurons/mm^2^ (49 ± 10 pyramidal cells/mm^2^) in BDNF-treated (*n* = 13 visual field per condition, four different series of cultures; *P* < 0.001, two-way ANOVA; Fig. 2b). This result suggests that BDNF treatment globally sustains neuronal survival and, in particular, that of excitatory pyramidal cells [49].

We then addressed whether these changes in network size and cellular composition were accompanied by changes in synapses. Accordingly, we recorded miniature EPSCs (mEPSCs; Fig. 2c) in a subset of Control and BDNF-treated neurons in the presence of the fast inactivating voltage-gated sodium channel blocker, tetrodotoxin (TTX, 1 μM), which impairs the action potentials. The analysis of mEPSCs allows assessing synaptic functional and structural components. In particular, mEPSCs reflect the stochastic release of vesicles from the presynaptic terminals and their frequency depends on the presynaptic release probability and on the number of synaptic contacts, whereas their amplitude depends on postsynaptic receptor [42, 52]. We found that mEPSC amplitude was increased by BDNF-treatments when compared to Control (from 30 ± 0.7 pA Control to 53.4 ± 2.0 pA BDNF-treated, *n* = 9 and *n* = 17, respectively). When plotting the cumulative probability distribution of mEPSCs peak amplitude values (Fig. 2d), a highly statistically significant difference (*P* < 0.001, Kolmogorov-Smirnov test) was detected between Control and BDNF-treated cultures. BDNF also significantly increased the frequency of mEPSCs (from 0.06 ± 0.02 Hz in Control to 0.40 ± 0.08 Hz in BDNF-treated neurons; P < 0.001, one-way ANOVA; bar plot in Fig. 2e). These results suggest that, upon BDNF treatment, both functional and structural synaptic components are affected.

### Long-term BDNF treatment affects functional components of synapses

**Fig. 3:**
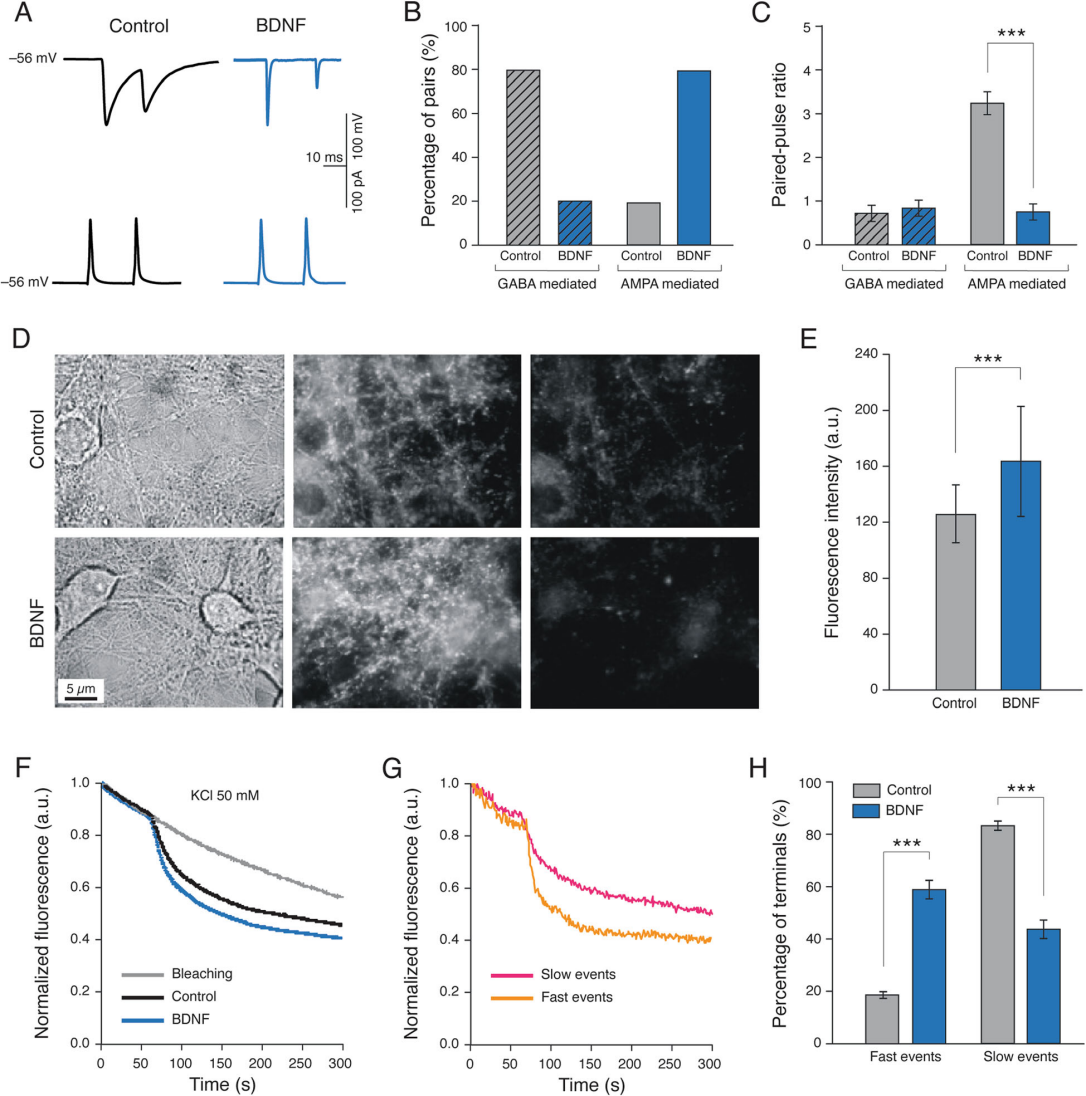
BDNF exposure affects pre-synaptic release. In (**a**) sample tracings of simultaneous pair recordings: top, evoked monosynaptic GABAergic PSCs in Control (black) and glutamate-AMPA receptor mediated in BDNF-treated (blue) cultures; bottom, presynaptic induced action potentials. In (**b**) plots summarize the probability of finding evoked GABA_A_- or AMPA- receptor mediated pair recordings in Control (grey) or BDNF (blue) treated cultures. In (**c**) is summarized the paired-pulse ratio (PPR) of GABA_A_- or AMPA-mediated PSCs measured in Control and BDNF treated pairs. In (**d**) sequence of images Control, above and BDNF, below: sequential FM1–43 staining and destaining: (left image) bright field images show the extensive neurite arborization that hippocampal neurons reach at 9÷10 DIV culture; (middle image): the same fields after staining with FM1–43; (right image): fluorescence images following destaining induced by 50 mM KCl. Remaining fluorescence represents non-specific staining. In (**e**) histograms summarized the averaged fluorescence intensity when loaded with FM1–43. BDNF-treated cells showed a significant increase (*P* < 0.001, Mann-Whitney test) compared to Control cultures. In (**f**) the rate of synaptic release from synaptic terminals, following KCl stimulation, in Control (black line) and BDNF (blue line) cultures. In grey the natural fluorescent bleaching after KCl stimulation. In (**g**) representative single de-staining fast (orange line) and slow (pink) profiles, based on their kinetics properties. In (**h**) plots summarize the distribution of fast and slow events in Control (grey) and BDNF-treated (blue) cultures. Note that in BDNF-treated cells prevailed fast events (on the left), while in Control cultures, the large majority displayed slow events (on the right)

To further inspect synaptic features in cultured neuronal networks, we exploited simultaneous dual patch-clamp recordings of mono-synaptically connected neurons. Action potentials were induced in the presynaptic neuron and the evoked postsynaptic unitary PSCs (delay 2 ms) were examined (see methods; representative traces in Fig. 3a). In these experiments, we detected only a small fraction of gap junctions-coupled neurons, in accordance with previously reported results [42]. Due to the low probability of finding gap-junction-coupled pairs, those detected were not further analysed. The percentage of cell pairs that exhibited a detectable unitary connection (coupled pairs) was strongly increased by BDNF treatments (from 20% in Control to 70% in BDNF-treated, *n* = 26 pairs and *n* = 38 pairs, respectively). We further evaluated the nature of mono-synaptic connections, by assessing the PSC decay, pharmacology and reversal potential. In BDNF-treated cultures, the large majority (80%) of mono-synaptically coupled pairs were AMPA receptor-mediated and evoked fast EPSCs (τ = 5.0 ± 0.6 ms) while in Control cultures, the 80% of pairs were GABA_A_ receptor mediated and evoked slow IPSCs (τ = 23.0 ± 0.8 ms). These results are summarized in the bar plot of Fig. 3b.

We turned then our attention to the presynaptic properties of glutamatergic unitary synapses by adopting paired-pulse stimulation protocols with short inter-stimulus interval (50 ms; Fig. 3a), where the second response can be either facilitated or depressed. At unitary synapses an increased probability of neurotransmitter release (*p*_*r*_) favours paired-pulse depression, whereas a decrease in the *p*_*r*_ favours facilitation [53–55]. We estimated the *p*_*r*_ by quantifying the paired-pulse ratio (PPR; see methods) [56, 57]. While the PPR values for unitary GABAergic couples were not affected by BDNF treatment (bar plot Fig. 3c), glutamate AMPA-receptor mediated couples exhibited in Control a PPR value of 3.2 ± 0.3 (*n* = 5 pairs; Fig. 3c) indicating the presence of a strong facilitation [42], while in BDNF-treated neurons the measured PPR of 0.7 ± 0.06 (*n* = 10 pairs) was expression of a depression at these synapses.

Altogether, these experiments strongly suggested an increased survival of glutamatergic (pyramidal) neurons and increased synaptogenesis, along with modulation of pre and postsynaptic components of glutamatergic connections upon BDNF long-term treatments. Therefore, we focused the next set of experiments on presynaptic modifications, in particular the *p*_*r*._ We measured the kinetics of synaptic vesicle release by real-time imaging of vesicles labelled with styryl dye FM1–43 dye [58–61] to monitor the rate of presynaptic vesicle recycling from hippocampal neurons treated or untreated by BDNF. In these experimental conditions we could not discriminate between GABAergic and glutamatergic neurons and synapses, however we focused on presynaptic terminal of presumed pyramidal neurons (see methods; Fig. 3d, left column). Following FM1–43 labelling, clusters of presynaptic puncta were visible as bright fluorescence spots (Fig. 3d, middle and right columns, at 2 different times). The effect of BDNF on the size of recycling pool of synaptic vesicles was estimated by measuring the raw fluorescence intensity of individual FM1–43 positive puncta, that is proportional to the number of vesicles endocytosed during synaptic vesicle recycling, following high KCl (50 mM [62];) depolarization, that is proportional to the number of vesicles endocytosed during synaptic vesicle recycling [58]. As shown in Fig. 3e, we detected a significant increase (*P* < 0.001, Mann-Whitney test) in puncta fluorescence intensity in BDNF-treated neurons (from 126.7 ± 35.7 a.u. in Control to 164.6 ± 42.4 a.u. in BDNF treated, mean ± SD, 459 and 409 puncta, respectively; *n* = 5 coverslips from 3 independent cultures for each condition), indicating that long-term treatment with BDNF increased the size of the pool of recycling vesicles. When analysing the decay time constant (τ; *n* = 459 terminals, Control; *n* = 425 terminals, BDNF-treated; *n* = 3 series of cultures) of the FM1–43 fluorescence de-staining profiles during vesicle exocytosis, we observed a difference in the kinetics displayed by Control and BDNF-treated synapses, as shown in Fig. 3f. In reference experiments, the image series captured on FM1–43 stained cells, but without the high- K^+^ de-staining stimulus, produced a baseline reference plot (see Fig. 3f, bleaching). By analysis of the kinetic of single de-staining events, we could identify two categories of responses (see example in Fig. 3g): “fast” events showing an initial (20 s) fluorescence drop fitting an exponential decay with τ of 13.27 ± 2.74 s (mean ± S.D., *n* = 133 terminals of 3 different experiments), and “slow” events displaying τ of 27.87 ± 3.96 s (*n* = 176 terminals of 3 different experiments; *P* < 0.001, two-way ANOVA). The two categories of events were differently distributed among Control and BDNF-treated samples, in that “fast” events prevailed on BDNF-treated cultures (57.8% ± 11.7 of the total events population; n = 425 terminals, variation from Control “fast” events with significance of *P* < 0.001 by two-way ANOVA test; Fig. 3h), while “slow” events predominated in Controls (83.4% ± 4 of the total events population; *n* = 459 terminals, difference from BDNF slow events with significance of *P* < 0.001, two-way ANOVA test; Fig. 3h).

### Investigating the role of intracellular BDNF overexpression

**Fig. 4:**
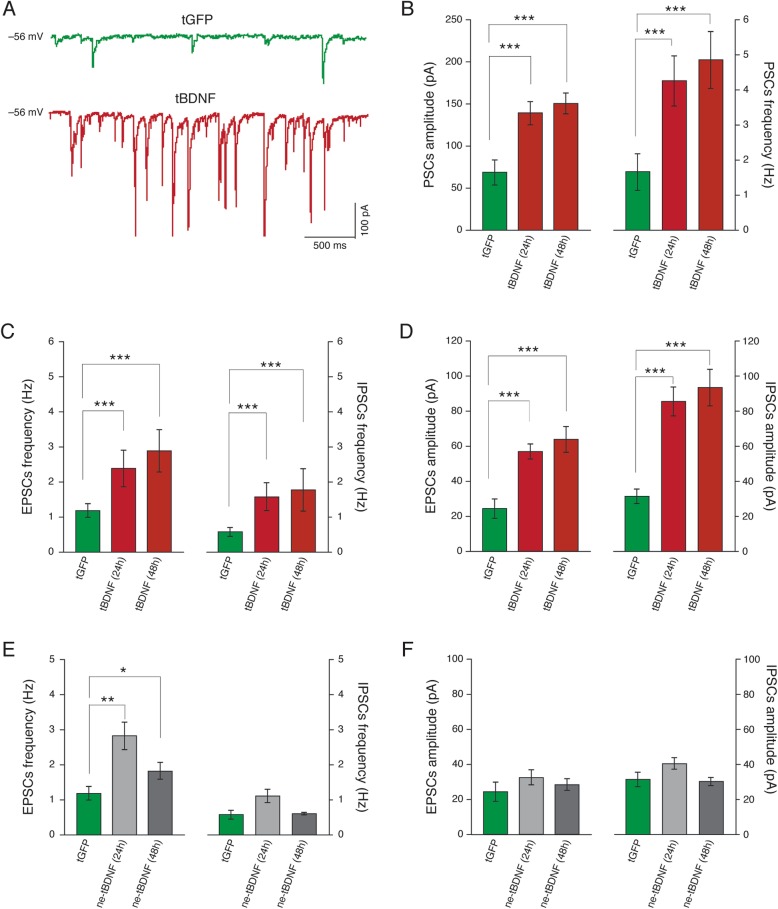
BDNF intracellular production and release affects all synaptic components. In (**a**) representative voltage clamp recordings of spontaneous activity in tGFP (green trace) and tBDNF (red trace; 24 h) cultures. In (**b**) plots reporting PSCs amplitude (left) and frequency (right), significantly (*P* < 0.001, two-way ANOVA) increased by BDNF expression (24 h and 48 h). In (**c**) plots reporting EPSCs (left) and IPSCs (right) frequency, significantly (*P* < 0.001, two-way ANOVA) increased by intracellular BDNF (24 h and 48 h) compared to Control sister cultures. In (**d**) plots reporting EPSCs (left) and IPSCs (right) amplitude significantly (*P* < 0.001, two-way ANOVA) increased by intracellular BDNF (24 h and 48 h) compared to Control sister cultures. In (**e**) and (**f**) EPSC and IPSC frequency and amplitude values are depicted recorded from ne-tBDNF. In respect of Control (tGFP) only EPSP frequency values were significantly affected (*P* < 0.01 24 h, *P* < 0.05 48 h, two-way ANOVA)

Since the results described above were obtained by treating neuronal cell cultures with exogenous BDNF, we designed a series of experiments to investigate the role of intracellular BDNF overexpression in modifying the activity of the cultured hippocampal network. Neurons at 8~10 DIV were recorded after 24 and 48 h from transfection with a plasmid encoding a specific BDNF-in fusion with GFP to allow identification of BDNF overexpressing cells. This construct (CDS-BDNF-GFP) is known to drive a local BDNF production and release in the cell body and in all proximal and distal compartments of dendrites [63–65]. By using voltage clamp recordings (Fig. 4a for sample tracings), we compared GFP transfected Control (*n* = 11, from now on tGFP) vs. CDS-BDNF-GFP transfected neurons (from now on tBDNF; 24 h tBDNF n = 11; 48 h tBDNF *n* = 8, 48 h). Passive membrane properties were not affected by BDNF production and release (tGFP capacitance 63 ± 7 pF, at 24 h tBDNF 53 ± 6 pF and at 48 h tBDNF 58 ± 6 pF; input resistance 210 ± 46 MΩ, 300 ± 84 MΩ and 270 ± 32 MΩ, respectively for tGFP, tBDNF 24 h, tBDNF 48 h).

In tBDNF neurons (24 h and 48 h), we detected a strong increase (*P* < 0.001, two-way ANOVA) in PSCs amplitude (70 ± 15 pA, in tGFP, 141 ± 15 pA in tBDNF 24 h and 152 ± 13 pA in tBDNF 48 h) and frequency (1.7 ± 0.5 Hz in tGFP, 4.3 ± 0.7 Hz in tBDNF 24 h and 4.9 ± 0.8 Hz in tBDNF 48 h), summarized in Fig. 4b. BDNF intracellular production and release affected all synaptic components, regardless of the transmitter involved. Figure 4c and d summarize these results, showing that in tBDNF (24 h and 48 h) both EPSCs and IPSCs were improved in amplitude and frequency. These results confirmed that the BDNF coding region, common to all transcripts [64] is sufficient to induce significant changes in the hippocampal synaptic transmission in cultures.

**Fig. 5:**
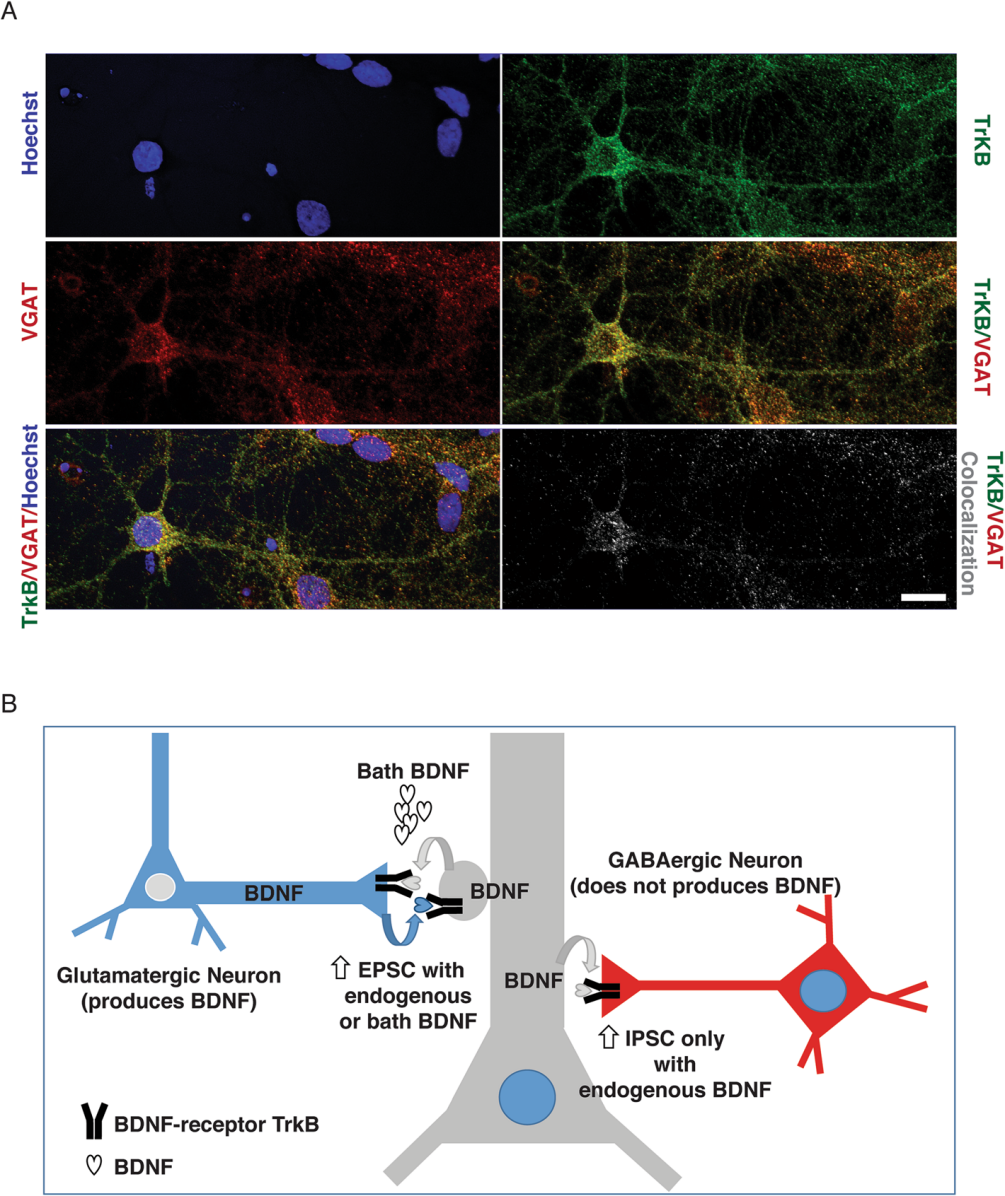
Colocalization of TrkB-full length receptor and VGAT. In (**a**) confocal images showing hippocampal neurons (Control culture) (anti-TrkB, in green; anti-VGAT, in red; nuclei are visualized by Hoechst in blue). Merge of the TrkB and VGAT channels or merge of the three channels are shown in dual o three-colour. The black and white image shows the colocalized TrkB and VGAT pixels. Note the presence of TrkB receptors at inhibitory synapses in the soma and the proximal domains of the apical and basal dendrites. Calibration bar: 10 μm. In (**b**) schematic summary of the proposed actions of BDNF when locally released at glutamatergic or GABAergic synapses

In the same set of experiments, we monitored the ability of intracellular over-expression of BDNF to impact neighbour cells (*n* = 11) not expressing CDS-BDNF-GFP (named ne-tBDNF). ne-tBDNF cells (distant ~ 100 μm from tBDNF ones; under visual microscope Control) were recorded at 24 h and 48 h, from the tBDNF cultures. ne-tBDNF cells displayed no differences in terms of capacitance and input resistance (50 ± 6 pF and 232 ± 28 MΩ, respectively). Figure 4e and f summarises results related to EPSCs and IPSCs frequency and amplitude values with respect to Control (tGFP): in ne-tBDNF we detected an increase in EPSCs frequency at 24 h and 48 h, without changes in EPSCs amplitude, while IPSCs parameters were not affected. These results suggest that localized production and secretion of BDNF can modulate pre and post synaptic activity locally, without effects on distant non-expressing neurons, besides those neurons reflecting the overall network increased activity. Eventually, we ascertained by confocal analysis, the presence of TrkB receptors at inhibitory synapses by co-labelling of cultured hippocampal neurons (Control untreated ones) for the TrkB receptor and for the VGAT, the vesicular GABA transporter considered a GABAergic presynaptic marker [42]. Figure 5 A qualitative analysis of the putative co-localization unequivocally demonstrated the presence of TrkB receptors at inhibitory synapses, distributed in the expected subcellular districts, namely the soma and the proximal domains of the apical and basal dendrites.

## Discussion

In this study, we show that prolonged (4 days-, 24 h- and 48 h-long) treatments with exogenous BDNF in DIV 4 hippocampal neurons, increased both amplitude and frequency of the excitatory synaptic activity recorded at DIV 8~10, leaving inhibitory synapses unchanged. Furthermore, we demonstrate that an intracellular overexpression of BDNF for 24 h or 48 h in hippocampal neurons increases both the amplitude and the frequency of the excitatory as well as inhibitory synaptic activity.

The targeting of glutamatergic synapses by BDNF is in agreement with previous studies [21, 35, 66–69]. We hypothesize that several converging mechanisms contributed to the up-regulation of excitatory synapses following chronic treatment with exogenous BDNF. We documented an increased survival of neurons in BDNF-treated cultures and, in particular, the doubling of survived pyramidal neurons, usually displaying a higher ratio in excitatory glutamatergic synapses than other cells [49]. The emergence of a larger network, characterized by an increased excitation/inhibition ratio, might contribute to the enhanced network activity [70]. Regardless the network size, we cannot rule out that a higher cellular excitability induced by BDNF [4] could be, at least in part, responsible for our results. Several pieces of experimental evidence suggest that a target refinement of structural and functional components of glutamatergic synapses took place during long-term exposure to the neurotrophin. Our results with miniature postsynaptic (PSC) recordings in TTX after BDNF treatment clearly support, in parallel with changes in the sensitivity of the postsynaptic cells to glutamate [26, 68, 71, 72], an enhancement in neurotransmitter release probability and in the number of releasing sites, in agreement with previous studies [73]. In particular, we show that BDNF treatment increases the probability of functional pairing between excitatory cells. The regulation of these presynaptic mechanisms is strengthened by our results in postsynaptic responses to paired-pulse stimulation, and by live imaging experiments, indicating variations in presynaptic transmitter release. Indeed, we found that BDNF induced an increased rate of FM1–43 release, leading to an increased percentage of fast release events with respect to Control cultures where slow ones prevail. Although the kinetics of styryl dye de-staining might account for multiple events, possibly comprising different steps of both exo- and endocytosis [74–76], these results reinforce the view that long-term treatment of hippocampal neuronal cultures with BDNF increases both the size and the availability of the releasable synaptic vesicle pool [68, 73]. In this framework, prolonged application of exogenous BDNF may also accelerate synapse maturation by inducing the expression of AMPA receptors and by stimulating their insertion in the plasmamembrane at the post-synaptic densities. Thus, an enhancement in mEPSC frequencies and amplitudes, as observed in our study, might also result from postsynaptic modifications; namely, the activation of postsynaptic silent glutamatergic synapses [16].

While the increased responsiveness of glutamatergic synapses with long-term bath application of BDNF is in perfect agreement with one previous study [69] we obtained novel findings with intracellular expression of BDNF-CDS.

In the present study, exogenous application of BDNF did not elicit significant changes in inhibitory PSCs. In previous studies, prolonged application of exogenous BDNF was shown to promote the formation/maturation of GABAergic synapses by presynaptic modifications [20, 21, 31, 68, 77], while evidence of post-synaptic effects, such as membrane expression of GABA_A_ receptors, are comparatively more limited [29, 78]. Several experimental differences concerning animal and culture ages may explain these contrasting results. First, in our study BDNF (20 nM) was bath-applied for 4 days from DIV 4 until DIV 8 (or DIV 6 to DIV 10) while in the other studies neurons were treated from DIV 1 for 1 or 2 weeks, at different BDNF concentrations ranging from 25÷100 ng/mL. Second, we used hippocampal neurons explanted from post-natal rats, while the other studies used E18 embryos [20, 21, 29, 31, 77, 78]. Of note, one study in which no effects on GABAergic synapse could be found [69] used DIV 12–14 primary cultures of hippocampal neurons treated with bath application of 100 ng/mL BDNF for 48 h. These discrepancies might be explained in the light of the effects of BDNF at different ages and concentrations. Young postmitotic neurons are very sensitive to BDNF dosage, while mature neurons are less sensitive and use different mechanism of response. In fact, intracellular overexpression of BDNF at early developmental stages induced raising of expression of K^+^/Cl^−^KCC2 co-transporter, which is responsible for the conversion of GABA responses from depolarizing to hyperpolarizing, and increased spontaneous co-active network activity, without altering the expression of GABA and glutamate ionotropic receptors [79]. In contrast, BDNF application on mature neurons is unable to affect synaptic vesicle release at inhibitory synapses while it increases spontaneous synaptic vesicle release at excitatory synapses [69]. We cannot exclude that other factors, such as low BDNF concentration ranges, when applied exogenously, influenced the synaptic outcomes of prolonged treatments. The concentration used in our work for exogenous BDNF applications is 20 nM, which is within the range of those reported by previous works (Reviewed in [80, 81]). Interestingly, our results are also in agreement with Shinoda et al. [69], where experiments were performed using a 48 h exposure with a lower concentration (~ 4 nM). Thus, apparently, using a concentration ~ 5 times higher did not affect the synaptic outcomes. It has been reported that acute or gradual increases in BDNF might influence downstream signaling [82], regardless TrkB being maximally phosphorylated in 5 min at 1 nM BDNF concentration. The possibility that, more than the final concentration of BDNF, is the mode of delivery which might impact its effects [82] cannot be excluded.

In a previous study, we have shown that the neuronal growth and maturation in vitro of both mouse and rat hippocampal neurons can be subdivided into six stages [44] which is a revision of the previous subdivision into 5 stages of the establishment of neuronal polarity, originally proposed by Dotti et al. [83] (reviewed in [84]). Thus, here we applied BDNF in a time window, which, according to the new staging system, spans the whole stage 4 (DIV 4÷6) and the beginning of stage 5 (DIV 7÷11) [44]**.** During Stage 4, neurons are still immature and primary dendrites assume a stable morphology while higher order dendrites and axons undergo progressive growth [44]. During Stage 5, apical dendrites undergo to highly dynamic series of protrusions and retractions [44, 85]. It is during Stage 5 that synapses become stabilized and the first, yet sparse, spontaneous action potentials are expressed [44].

Regardless the potential relationship between developmental stages and BDNF preferred targeting of excitatory synapses, we found here that the delivery route might impact synaptic targeting. We observed, for the first time, that intracellular overexpression of BDNF for 24 and 48 h within the developmental stage 5 elicited not only a significant increase in amplitude and frequency of excitatory PSCs but also of inhibitory ones.

Although we did not further investigate synaptic changes due to BDNF over expression, we put forward the provocative hypothesis that the BDNF delivery route is crucial to achieve postsynaptic effects. Several investigations have provided definitive evidence that transfection of a plasmid encoding GFP-tagged BDNF coding sequence leads to expression of the chimeric mRNA in the neuronal soma and dendrites, but also to transport of the GFP-tagged protein from the soma into dendrites and axons [63, 65, 86–89]. In particular, in neurons transfected with this plasmid, the overexpressed GFP-tagged BDNF can be locally translated from mRNA localized in the dendritic compartment, in proximity to synapses leading to local secretion and spatially restricted activation of TrkB receptors [64]. Interestingly, the results obtained from these in vitro studies correspond to the BDNF-GFP distribution observed in a recent in vivo study using a knock-in mouse [90]. The reason is that the coding sequence of BDNF contains constitutively active dendritic targeting signals, recruiting Translin and other RNA-binding proteins, that are sufficient to induce localization of BDNF mRNA in distal dendrites in unstimulated neuronal cultures [63], while the 3’UTR short and the 3’UTR long of BDNF contains signals that induce enhanced dendritic targeting of BDNF mRNA in response to neuronal activity [91]. Therefore, we can assume that even in this work, BDNF-GFP proteins are actually synthesized in the soma and in the dendrites. This localized production and secretion of BDNF from postsynaptic sites, is postulated to produce specific punctual effects on the synapses that are releasing this neurotrophin both through an autocrine loop impacting on the dendrite itself, as well as through a retrograde signalling affecting presynaptic terminal ([65, 92]; scheme in Fig. 5b). In our study, this potential mechanism is reinforced by the recordings from ne-tBDNF distinct neurons and by the detected co-localization of TrkB receptors and VGAT positive presynaptic terminals. An impairment of this mechanism has been associated with memory deficits in mice and humans [93]. Another mechanism which may underlay the differences observed between exogenous and endogenous long-term BDNF application, may involve the ability of glial cells, in particular astrocytes, to uptake circulating BDNF [94, 95], thus leading to different effects.

## Conclusions

In conclusion, the results of the present study suggest that intracellular overexpression of BDNF in developing neurons, provides a more complete and physiological stimulus to induce maturation of excitatory and inhibitory synapses, with respect to exogenous application of BDNF. Previous attempts to use intravenous or intrathecal delivery of exogenous BDNF for the treatment of neurodegenerative diseases have failed [96, 97]. Thus, current therapeutic approaches have been redirected to delivery of mesenchymal cells overexpressing BDNF [98] or to viral-mediated gene therapy in the CNS parenchima [99]. In this framework we perceived as relevant to provide a detailed analysis of the differential synaptic outcomes of BDNF treatments through bath application vs genetic delivery in cultured neuronal networks.

## Methods

### Preparation of primary cultures

Dissociated rat hippocampal cultures were prepared from 2- to 3-days postnatal (P_2_-P_3_) animals as previously reported [42, 48]. All procedures were approved by the local veterinary authorities and performed in accordance with the Italian law (decree 26/14) and the UE guidelines (86/609/CE, 2007/526/CE, and 2010/63/UE). The animal use was approved by the Italian Ministry of Health. All efforts were made to minimize suffering and to reduce the number of animals used. All chemicals were purchased by Sigma-Aldrich unless stated otherwise. Enzymatically dissociated cells [42, 48] were plated at a density of 200.000 ± 16.000 cells/mL (values sampled form *n* = 4 cultures) on poly-L-ornithine-coated glass coverslips (Kindler, EU) in 35 mm Petri dishes. Cultures were incubated in a 5% CO_2_ humidified incubator in medium MEM supplemented to reach 35 mM glucose, 1 mM Apo-transferrin, 15 mM HEPES, 48 μM insulin, 3 μM biotin, 1 mM vitamin B_12_, 500 nM gentamicin, and 10% dialyzed foetal bovine serum (FBS; Gibco). Culture medium was renewed after 2 days from seeding and contained additionally an inhibitor of glial cells proliferation, cytosine arabinoside (Ara C, 10 μM). After 4 days in vitro, cultures were treated for a further 24 h, 48 h or 4 days with 20 nM Brain-Derived Neurotrophic Factor (BDNF). Cultured cells were used for experiments at 8~10 days in vitro.

### Electrophysiological recordings

Single and paired patch-clamp recordings in the whole-cell configuration were obtained at room temperature (RT) with glass pipettes (5–7 MΩ) containing (in mM): 120 K gluconate, 20 KCl, 10 HEPES, 10 EGTA, 2 MgCl_2_, 2 Na_2_ATP, pH 7.3; osmolarity was adjust to 300 mOsm. The extracellular solution contained (in mM): 150 NaCl, 4 KCl, 1 MgCl_2_, 2 CaCl_2_, 1 MgCl_2_, 10 HEPES, 10 glucose, pH 7.4. Either an EPC-7 amplifier (List, Germany) or a Multiclamp 700B (Axon CNS, Molecular Devices) patch amplifier were used for voltage clamp recordings with the cell voltage clamped to − 56 mV holding potential (not corrected for junction potential, that was 14 mV). For current clamp recordings we used an Axoclamp 2B amplifier (Molecular Devices LLC, Axon Instrument, US) or a Multiclamp 700B under bridge-balance mode. Current and voltage clamp responses were digitized using a Digidata 1322A or a Digidata 1440A (Molecular Devices LLC, US) at 10 KHz with the pClamp 10.2 acquisition software (Molecular Devices LLC, USA) and stored for further analysis. In paired recordings, the presynaptic neuron was held under current clamp mode at − 70 mV (≤0.02 nA negative current injection), and action potentials were elicited by injecting short (4 ms) square current pulses (1 nA). The postsynaptic cell was voltage clamped at − 56 mV holding potential. Monosynaptic connections were recognized by their short latency (< 5 ms [100];), measured between the peak of the evoked action potential and the onset of the postsynaptic current response. We addressed the presence of gap-junctions by delivering hyperpolarizing current steps (− 0.05 nA impulses, 100 ms in duration [101];). All recorded events were analyzed offline with the AxoGraph 1.4.4 (Axon Instrument) event detection software (Axon CNS, Molecular Devices). To characterize the short-term dynamics of synaptic contacts, we delivered to pairs of connected neurons (*n* = 6 Control and *n* = 10 BDNF-treated) paired pulse stimulations at 20 Hz (every 20 s; 10 times that were pooled together and averaged [48];). We measured the paired-pulse ratio (PPR, calculating the ratio between the mean peak amplitude of the second and the first PSC [48]).

### Immunofluorescence staining

Hippocampal cells, Control and BDNF-treated, were fixed by 4% formaldehyde (prepared from fresh paraformaldehyde; Sigma) in PBS at RT for 20 min. They were permeabilized with 0.1% Triton X-100 and blocked in 1% foetal bovine serum (FBS) in PBS for 30 min to prevent unspecific binding of antibodies. After incubation with the primary antibodies (rabbit polyclonal anti-β-tubulin III, 1:500 dilution; mouse monoclonal anti-GFAP, 1:500 dilution) for 1 h at RT, cells were incubated for 30 min with the secondary antibodies Alexa Fluor 594 goat anti-rabbit (Invitrogen, 1:500 dilution), AlexaFluor 488 goat anti-mouse (Invitrogen, 1:500 dilution) and with DAPI (Invitrogen, 1:200 dilution) to stain the nuclei. Samples were mounted in Vectashield (Vector Laboratories) on 1 mm thick coverslips. Cell density was quantified at 20 × magnification using a Nikon Eclipse Ti-U fluorescence microscope with random sampling of ten fields (Control and BDNF treated, *n* = 4 culture series each). Neuronal densities were calculated for the central area and for the periphery (distal areas) of the glass coverslip by manual counting after the calibration of the microscope field of view. The shape of the soma and the processes organization and number were used to identify pyramidal neurons [49]. Specifically, cells with a triangular soma of 12–18 μm dimeter and with at least one thick process were assumed to be pyramidal [49, 51]. Offline analysis was performed with the image-processing package Fiji [102].

### Colocalization of TrkB-full length receptor and VGAT

Unstimulated Control neurons were permeabilized with PBS/0.1% Triton-X100, blocked with 1% BSA in PBS/0.1%Triton-X100 and co-stained for 1.5 h with 1:500 anti-TrkB (R&D Lab) (secondary 1:200 anti-goat Alexa-488 in green) and with 1:250 (mouse) monoclonal antibody against the vesicular GABA transporter (VGAT) anti-VGAT antibodies (Synaptic Systems) (secondary anti-mouse Alexa-568 in red) in PBS/0.1% Triton-X100. Nuclei were labelled by 5 min incubation with Hoechst/PBS (in blue).

Images were taken with a 60 × oil immersion objective on a Nikon C1si spectral Confocal microscope and deconvoluted using the standard protocol of Huygens 19.1 (Scientific Volume Imaging BV, Hilversum-The Netherlands). Merge of the TrkB and VGAT channels or merge of the three channels are shown in dual o three-colour. The black and white image shows the colocalized TrkB and VGAT pixels extracted with Huygens. The qualitative analysis indicates the presence of TrkB receptors at inhibitory synapses mostly located in the soma and the proximal domains of the apical and basal dendrites. Co-localization extends into secondary and in part tertiary dendrites, but is very rare in distal dendritic regions which are known to be characterized by low density of inhibitory synapses.

### FM1–43 fluorescence imaging

Depolarization-dependent staining of synaptic terminals with the styryl dye N-(3-triethylammoniumpropyl)-4-(4-(dibutylamino) styryl) pyridinium dibromide (FM1–43, Molecular probes, Life Technologies Corporation) was obtained by incubating cultures (after 10 min saline buffer wash at RT) for 120 s with 2 mL of saline solution containing 50 mM KCl and 15 μM FM1–43 dye. The buffer was replaced with 2 mL of normal saline solution containing FM1–43, and cells were left to recovery for 10 min to ensure complete recycling of the vesicles and then incubated for 10 min with saline containing 10 μM 6-cyano-7-nitroquinoxaline-2,3-dione (CNQX) and 50 μM 2-aminophosphonovaleric acid (2-APV) to prevent network activity altering the rate of FM release. These antagonists were present throughout the experiment. Images were continuously acquired by a Till Photonics Till-Imago system, exciting the FM1–43 dye with a 475 nm wavelength generated by a monochromator (Polychrome IV, Till Photonics GMBH, Grafelfing, Germany) and acquiring fluorescence images using a 800 × 600 pixels CCD camera (CCD Imago type super VGA, Till Photonics; 1 frame/second, 50 ms exposure) interfaced to the TillVision software (Till Photonics). Application of 50 mM KCl (2 min), followed by a 2 min washout, was used to stimulate vesicle endocytosis from the dye-containing terminals, measured as a fluorescence loss. Offline analyses were performed on image sequences with TillVision software. Time-dependent fluorescence changes on FM1–43 labelled terminals were obtained by drawing regions of interest (ROIs) around fluorescent spots (typically 6 × 6 pixels), including as little background possible. A comparison of the brightness of total vesicle pool puncta (raw fluorescent intensity) in BDNF-treated and Control cultures before the unloading stimulus gave an estimate of the number of vesicles endocytosed during FM1–43 loading. The decay time costant, τ, was measured using Origin Pro 7.5 sofware (OriginLab Co., US).

### Plasmid preparation and transfection

Plasmids were previously described [63] and contained a preproBDNF-GFP construct inserted in a pEGFP-N1 vector (Clontech). Primary hippocampal neurons were transfected with Lipofectamine 2000™ (Life Technology, Invitrogen) [103]. Cells were transfected with 1 μg of plasmid DNA diluted in 50 μL of MEM medium without serum and antibiotics. At the same time 2 μL of the Lipofectamine™ solution (1 mg/mL) have been dispersed in 50 μL of MEM solution. After 5 min the two solutions have been mixed together, let for 20 min stabilizing at RT, diluted to a final concentration of 20 nM and then added to the cellular culture medium for transfection. After 1 h incubation the Lipofectamine-DNA mixture has been carefully removed and replaced with culture medium. GFP-alone transfected neurons were used as Control.

### Statistical analysis

All values from samples subjected to the same experimental protocols were pooled together and results are presented as mean ± S.D., if not otherwise indicated; n = number of cells, if not otherwise indicated. All data were compared using parametric and nonparametric tests (Student’s *t* test, Mann-Whitney or Kolmogorov-Smirnov test) as well as one-way and two-way ANOVA, adjusting comparison by Bonferroni or Holm-Sidak correction. Statistical significance was determining at *P* < 0.05.

## Data Availability

The datasets supporting the conclusion of this article are included within the article. The datasets generated and/or analysed during the current study are stored in a public repository and are available from the corresponding author on reasonable request.

## Abbreviations

AMPA: α-amino-3-hydroxy-5-methyl-4-isoxazolepropionic acid
BDNF: Brain derived neurotrophic factor
CNS: Central nervous system
EPSCs: AMPA-receptor mediated excitatory post-synaptic currents
GABA: ɣ-Aminobutyric acid
GFAP: Glial fibrillary acidic protein
IPSCs: GABA_A_-receptor mediated inhibitory post-synaptic currents
mEPSCs: AMPA-receptor mediated miniature excitatory post-synaptic currents
PPR: Paired-pulse ratio
*P*_*r*_: Probability of neurotransmitter release
PSCs: Postsynaptic currents
RT: Room temperature (RT)
TTX: Tetrodotoxin
VGAT: Vesicular GABA transporter
